# Mesoporous Silica Microparticle-Protein Complexes: Effects of Protein Size and Solvent Properties on Diffusion and Loading Efficiency

**DOI:** 10.3389/bjbs.2024.13595

**Published:** 2024-10-09

**Authors:** Mohamad Anas Al Tahan, Kyprianos Michaelides, Smith Somasekharan Nair, Shouq AlShatti, Craig Russell, Ali Al-Khattawi

**Affiliations:** ^1^ School of Pharmacy, College of Health and Life Sciences, Aston University, Birmingham, United Kingdom; ^2^ Aston Medical Research Institute, College of Health and Life Sciences, Aston University, Birmingham, United Kingdom

**Keywords:** mesoporous silica, microparticles, protein oral delivery, diffusion, fluorescence intensity

## Abstract

Oral administration of protein-based therapeutics is highly desirable due to lower cost, enhanced patient compliance, and convenience. However, the harsh pH environment of the gastrointestinal tract poses significant challenges. Silica-based carriers have emerged as potential candidates for the delivery of protein molecules, owing to their tuneable surface area and pore volume. We explored the use of a commercial mesoporous silica carrier, SYLOID, for the delivery of octreotide and bovine serum albumin (BSA) using a solvent evaporation method in three different solvents. The loading of proteins into SYLOID was driven by diffusion, as described by the Stokes-Einstein equation. Various parameters were investigated, such as protein size, diffusion, and solubility. Additionally, 3D fluorescence confocal imaging was employed to identify fluorescence intensity and protein diffusion within the carrier. Our results indicated that the loading process was influenced by the molecular size of the protein as octreotide exhibited a higher recovery rate (71%) compared to BSA (32%). The methanol-based loading of octreotide showed uniform diffusion into the silica carrier, whereas water and ethanol loading resulted in the drug being concentrated on the surface, as shown by confocal imaging, and further confirmed by scanning electron microscopy (SEM). Pore volume assessment supported these findings, showing that octreotide loaded with methanol had a low pore volume (1.2 cc/g). On the other hand, BSA loading was affected by its solubility in the three solvents, its tendency to aggregate, and its low solubility in ethanol and methanol, which resulted in dispersed particle sizes of 223 and 231 μm, respectively. This reduced diffusion into the carrier, as confirmed by fluorescence intensity and diffusivity values. This study underscores the importance of protein size, solvent properties, and diffusion characteristics when using porous carriers for protein delivery. Understanding these factors allows for the development of more effective oral protein-based therapeutics by enhancing loading efficiency. This, in turn, will lead to advances in targeted drug delivery and improved patient outcomes.

## Introduction

Researchers and pharmaceutical companies have long sought oral delivery of peptides/proteins due to its enhanced patient compliance, cost-effectiveness, and non-invasiveness/convenience compared to injection-based delivery [1]. However, there are many barriers to the oral administration of biomolecules, including pre-absorption challenges, namely degradation due to both pH differences in the gastrointestinal tract (GI tract) and enzymatic activity, which cause lower bioavailability of orally administered products [2, 3]. In addition, the limited absorption across the intestinal epithelium represents a major barrier, as large molecules with lipophilic properties generally crossing the intestinal barrier via the transcellular route, whereas hydrophilic molecules cross via the paracellular route involving through the tight junctions [4].

Some of the previous barriers have been successfully overcome, resulting in several FDA-approved products like Rybelsus^®^ and Mycapssa^®^ for the oral delivery of semaglutide and octreotide acetate, respectively [5]. Both products were manufactured using a novel technology that utilises novel compounds called permeation enhancers to facilitate the transport of the drug molecules across the intestinal epithelium [6].

In addition to existing technologies, pharmaceutical companies and formulation scientists are searching for versatile, generally recognised as safe (GRAS), and biocompatible materials, with mesoporous silica falling into this category while being approved by the FDA [7]. They possess several advantages that make them sought after for peptide delivery, such as large surface area, tuneable pore size and high pore volume [8]. The International Union of Pure and Applied Chemistry (IUPAC) has established a classification for porous materials that includes mesoporous silica [9].

There are two general categories for mesoporous silica loading strategies: solvent-free and solvent-based methods. The first includes physical mixing, melting, co-milling, and microwave irradiation. The second consists of adsorption, solvent evaporation, incipient wet impregnation, supercritical fluid technology, diffusion-supported technology, covalent grafting, co-spray drying, and chaperone assistance [7, 10, 11]. Regardless of the loading approach, mesoporous silica protects peptides from enzymatic degradation if they remain within the pores [12]. Furthermore, they protect biomolecules from bacterial decomposition degradation contribute to formulation flexibility, making them good candidates for biomolecule loading [13].

However, many factors affect the efficiency of protein loading on mesoporous silica. For instance, choosing a suitable solvent is one of the most important factors. It has been reported that using a polar solvent will reduce the loading of a hydrophobic active pharmaceutical ingredient (API), as it will compete to interact with the silica surfaces [14]. Moreover, non-polar solvents are more suitable for loading hydrophobic APIs into mesoporous silica nanoparticles due to the formation of hydrogen bonds between the drug molecules and silanol groups [15].

In terms of protein properties, loading is strongly affected by molecular size and surface charge. Small molecular weight proteins can occupy the pores better than high molecular weight ones, as small proteins can penetrate the internal surfaces, while large proteins are limited to the external surfaces [16]. In terms of molecular charge, the surface of silica carriers is covered with Si-OH groups, which usually act as adsorption sites and provide the carrier with a negative charge. This facilitates the interaction with positively charged molecules based on electrostatic interactions. Nevertheless, negatively charged molecules can be loaded with higher efficiency when a chaotropic agent is applied, if it screens the repulsion and allows higher loading [17].

**FIGURE 1:**
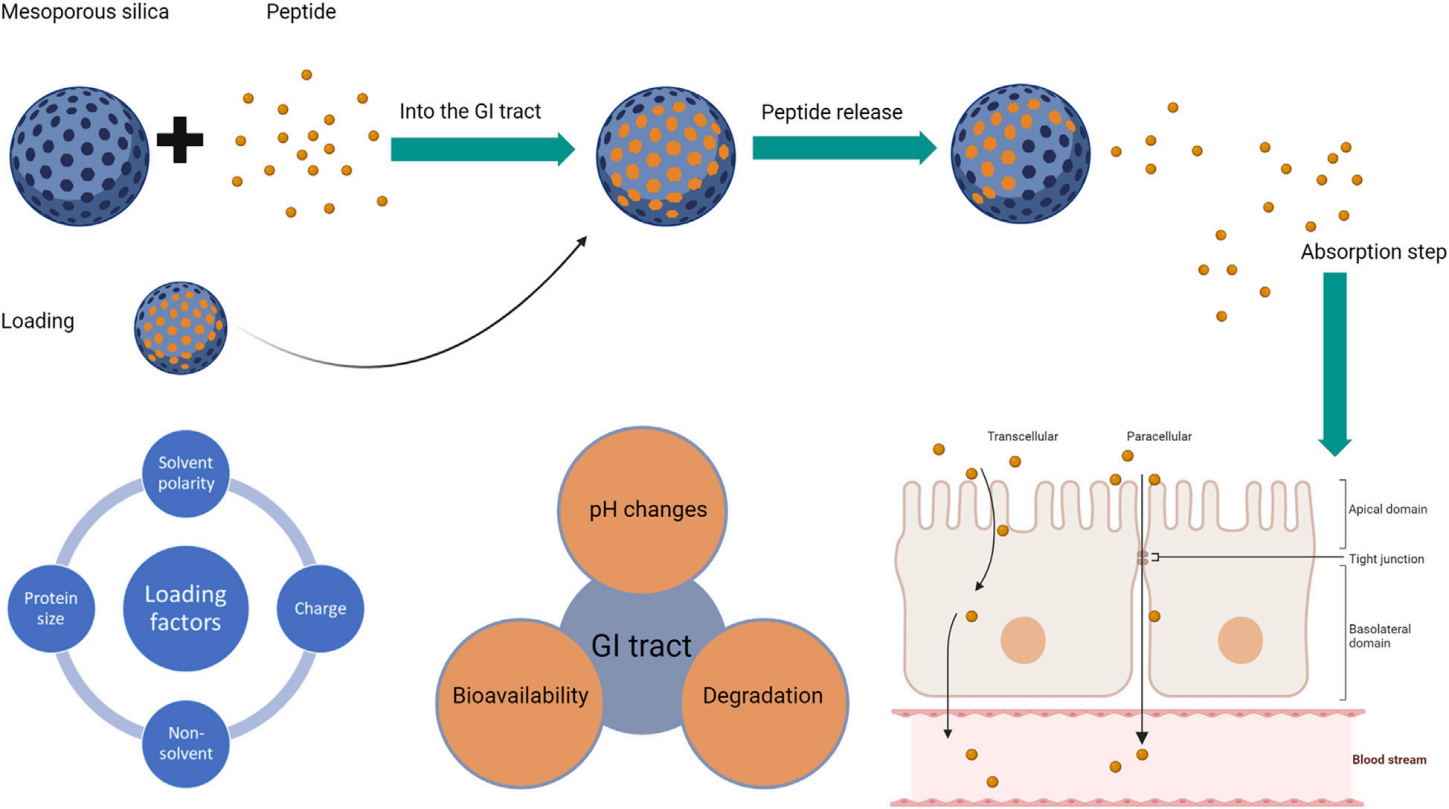
The process of successful oral delivery if a peptide-silica complex with the factors affecting each step: factors affecting silica loading (left), followed by factors affecting the peptide (middle), and the absorption routes for peptides whether transcellular or paracellular (right). The blue sphere represents the silica carrier, while the small orange circle is the loaded peptide.

The process that affects the successful oral delivery of a peptide-silica complex is summarised in Figure 1 below based on the process of formulation and administration.

This study aims to investigate the factors that affect the mesoporous silica loading of bovine serum albumin (BSA) and octreotide acetate, to better understand how size, solvent properties, and protein concentration can affect the loading efficiency. BSA is a large protein containing approximately 58 amino acids with a molecular weight of approximately 68 kDa [18]. Octreotide acetate is a hydrophilic octapeptide with 8 amino acids and has a molecular weight of approximately 1 kDa [19]. The chemical structures of both proteins are highlighted in Supplementary Figure S1. This will involve loading both proteins via three solvents at different concentrations (5%–20% w/w) and quantifying the actual drug load via HPLC. Fluorescence imaging will be implemented to further understand the loading based on diffusivity, while the morphology will be checked by scanning electron microscopy.

## Materials and Methods

### Materials

Ethanol absolute 99.8% (HPLC grade), methanol absolute 99.9% (HPLC grade), acetonitrile 99.8% (HPLC grade), and Bovine serum albumin (BSA) fraction V molecular weight 68 kDa were purchased from Sigma-Aldrich (Dorset, UK). Octreotide acetate (octreotide) lyophilised powder with a molecular weight of 1079.29 Da was purchased from Stratech (Cambridgeshire, UK). Polypropylene containers were purchased from Agar Scientific (Essex, UK). Mesoporous silica SYLOID XDP 3050 (specific surface area of 310 m^2^/g, average pore size of 22.4 nm, pore volume of 1.74 cm^3^/g) was kindly provided by W.R. Grace and Co. (Worms, Germany).

### Methods

#### Preparation of Mesoporous Peptide-Silica Complex Formulations

The preparation of peptide-mesoporous microparticles was conducted by the solvent evaporation method using three solvents; water, ethanol, and methanol. Peptides were dissolved in the solvent at concentrations ranging from 0.5 to 2 mg/mL, followed by the addition of SYLOID XDP 3050 to the mixture. The mixture was left on a magnetic stirrer for 2 h while covered. The suspension mixture was transferred to a watch glass and left in a fume hood for the solvents to evaporate. After solvent evaporation, the samples were kept in plastic containers for post-formulation characterisation. The theoretical loading of both peptides on SYLOID was 5, 10, and 20% w/w.

#### Drug Loading Quantification and Recovery

The actual drug load was calculated by adding 5 mg of the peptide-silica complex to 10 mL of distilled water in a polypropylene plastic container and leaving it on a magnetic stirrer for 2 h. Samples were taken from the container after 2 h and filtered using a 0.2 syringe filter into HPLC vials for analysis, and the remaining contents of the container were discarded. The presented results are based on the recovery percentage.

#### HPLC Methods for Analysis

The HPLC analysis for octreotide was adapted from [20] and conducted using an Agilent 1200 series at a UV wavelength of 280 nm. The column was a Gemini C18 (Phenomenex) with the following specifications: 150 mm, 4.5 mm, 5 μm, 110 Å. The injection volume was 50 μL at a flow rate of 0.8 mL/min. Mobile phases were: A (water + 0.1% TFA) and B (acetonitrile + 0.1% TFA) with an analysis run time of 6 min. The concentration for the mobile phases started at 70:30 A:B and decreased to 55:45 A:B at 3.6 min. Then, the concentration increased again to 70:30 A:B.

The BSA analysis was adapted from [21] and was conducted using a WATERS 2695 with excitation and emission wavelengths of 280 and 350 nm, respectively, and an injection volume of 100 μL. The column was a Jupiter*®* C5 with the following specifications 5 μm, 300 Å, 4.6 mm, and 250 mm. The mobile phases were A (water + 0.1% TFA) and B (acetonitrile + 0.1% TFA) at a flow rate of 1 mL/min, and the gradient was as follows: A:B from 95:5 to 35:65 in 20 min, followed by a 2-min recovery to initial conditions. Both HPLC methods were validated according to ICH Q2R2 guidelines [22].

#### Solvent and Loading Phase Viscosity Calculation

The viscosity of the loading solvents and the loading phase (solvent-protein) was calculated using a MicroVISC microviscometer (RheoSence, United States). 50 μL Samples were taken using microVISC disposable pipettes (100 μL), and the chip used was A05 (microVISC Chip, 0–100 cP 50 µm flow channel). Sample runs were conducted at room temperature, with a cleaning cycle running after each formulation to rinse and dissolve any particulates. Each sample was run in triplicate, and results are presented as Mean (mPa.s) ± SD.

#### Fourier-Transform Infrared Spectroscopy (FTIR) Analysis

The investigation of molecular interactions was assessed using a Nicolet™ iS™ 5 FTIR (Thermofisher, Waltham, United States) equipped with an ID5 diamond attenuated total reflectance (ATR) accessory. Before scanning, a background scan was collected, then approximately 30 mg of powder was placed on the diamond plate, and the spectrum was obtained by taking 36 scans in the 500–3,500 cm^−1^ region at 4 cm^−1^ resolution. Atmospheric suppression and advanced ATR corrections were implemented after scanning. OMNIC™ was used for analysing the spectra.

#### Protein Fluorescence Intensity Identification

Confocal microscopy (TCS SP8, Leica Microsystems, GmbH) was used for imaging. A 405 nm diode laser and a white light laser at 70% power were used to image the fluorophores AlexaFluor 405 and AlexaFluor 532 for octreotide and BSA, respectively. The excitation and emission wavelengths were 385 and 562 nm for octreotide, while they were 520–739 nm for BSA. For each channel, HyD detectors were used. A 20X dry APO lens was used for imaging, and all images were taken at 4,096*4,096 resolution with imaging speed set at 100 Hz. Laser power, gain, and emission wavelength were kept constant for quantification purposes. A 3D image of the particle was then taken, and the fluorescence intensity of the middle plane was calculated. The particle was divided into four sections, and the highest intensity value for each section was taken. In total, 10 particles were imaged for fluorescence intensity, and the mean values were used to determine the intensity value. For image analysis, LAS X 3.0 (Leica Microsystems GmbH) and Fiji [23] software were used.

#### Morphological Properties of the Formulations

Images were taken using the environmental scanning electron microscope mode of the ThermoFisher Scientific Quattro S microscope equipped with a field emission filament (FEG). Images were taken in low vacuum mode at variable pressure between 85 and 105 Pa, with an acceleration voltage of 3 kV and a spot size of 3–3.5.

#### Particle Size Analysis of Protein Dispersions

Sympatec laser diffraction (Germany) was used to determine the particle size of the non-dissolved protein dispersions in loading solvents (ethanol and methanol). The instrument was equipped with a HELOS/BR compact laser diffraction sensor and a CUVETTE wet dispersion system. The CUVETTE 50 mL extension was used, and the R5 Fourier lens (f = 100 mm) had a measuring range between 0.5 and 875 µm. A 30 mL sample was used, and the run started at a trigger condition of ≥ 1% Copt (optical concentration) for 100 ms, where it was sonicated for 5 s at 100% power and stirred at 1200 rpm throughout the analysis. The median diameter (VMD) was recorded for all samples, with all measurements performed in triplicate and reported as VMD (μm) ± SD. PAQXOS 5.0 was employed for result analysis.

#### Dynamic Light Scattering (DLS) Analysis

For size analysis of protein dissolved in the loading solvents, Dynamic light scattering (DLS) measurements were performed using the NanoBrook Omni from Brookhaven Instruments Corporation (Holtsville, NY, United States). Eppendorf disposable plastic cuvettes (Mississauga, ON, Canada) were used for the analysis. The apparatus was set at an angle of 90° at 25°C and the measurement time was 120 s with three measurements taken from each sample, and values were reported as Mean Effective Diameter (nm) ± SD.

#### Surface Area and Pore Volume Evaluation

The specific surface area was determined using the standard BET (Brunauer, Emmett, Teller) theory, using NOVAtouch LX2, Quantachrome instruments (Anton Parr, United States) with Nitrogen adsorption at 1 bar nitrogen gas purge at a relative pressure p/p_0_ in the range 0.05–0.3. The pore volume was determined using the BJH (Barrett, Joyner, and Halenda) adsorption and BJH desorption methods. The Kurk-Jaroniec-Sayari model [24] was used to calculate the pore size distribution of the carrier, as it was considered suitable for mesoporous silica carriers. Touchwin software (version 1.2 x) was used for data acquisition.

#### Statistical Analysis

Statistical data analysis was performed with SPSS 28 using one-way Analysis of Variance (ANOVA) coupled with a Tukey post-hoc test, with all experiments conducted in triplicate. The data are presented as mean ± SD, and a P-value < 0.05 is considered statistically significant.

## Results and Discussion

### Quantification of Actual Drug Load and Recovery

**FIGURE 2:**
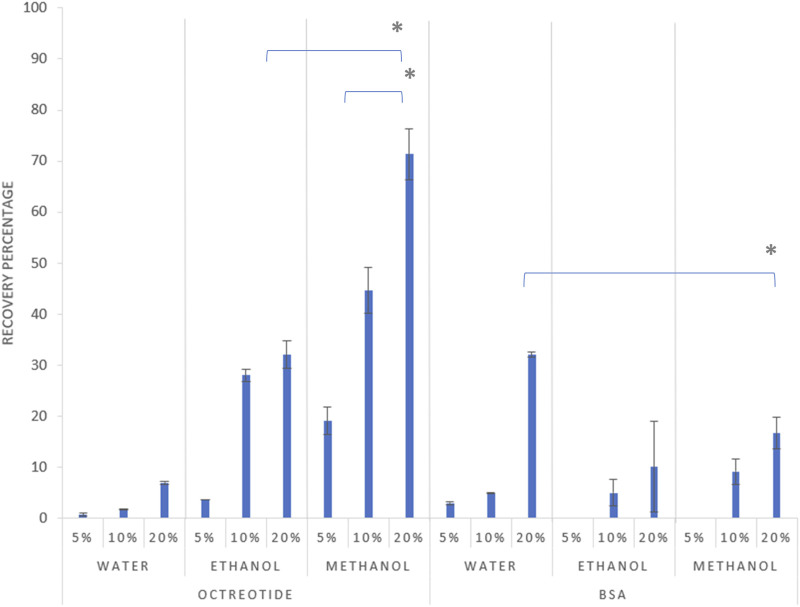
Recovery percentage of the formulations after 2 h at different theoretical loadings, 5%, 10%, and 20% drug load respectively for each solvent, with error bars representing the standard deviation and (*) representing a significant difference (p < 0.05).

The quantification of the actual drug load of both molecules in their respective solvents was investigated via HPLC, where the recovery percentage of the octreotide and BSA silica complexes was determined (Figure 2).

As shown in Figure 2 above, the recovery percentage of octreotide increased with increasing drug concentration in the loading solvents, with the lowest recovery associated with water at the initial loading concentration of 5% (w/w). However, upon using methanol as the loading solvent, recoveries were 19%, 44%, and 71% for concentrations of 5%, 10% and 20% w/w, respectively, as the higher concentration significantly increased the recovery percentage (p < 0.05). Moreover, ethanol presented a similar pattern for the relationship between loading concentration and recovery, with the highest recovery being 32% when the loading concentration was 20%. These differences are related to the difference in solubility of octreotide in the three solvents, which is addressed in *Effects of solvent polarity and protein solubility on loading*.

For BSA, the recovery appeared lower in comparison to the octreotide results, as the highest achieved recovery (32%) was associated with the sample being dissolved in water at a concentration of 20% w/w. The recovery of BSA in the three solvents followed a similar pattern to octreotide, where the recovery percentage increased with increasing loading concentration, which significantly increased the recovery percentage (p < 0.05). However, upon using methanol and ethanol as loading solvents, the recovery was 0% at an initial loading concentration of 5%, while the highest recovery values for both solvents were 16% and 10% for methanol and ethanol, respectively. The differences in recovery were related to several factors including solvent properties, the molecular size of the API and its solubility in the loading solvent, and diffusion-based factors.

### Effects of Solvent Polarity and Protein Solubility on Loading

The solvent used in active pharmaceutical ingredient (API) loading plays a crucial role during formulation, as the solvent properties dictate the interactions between the solute and the silanol groups on the surface of the carrier [25].

The solvents used have different polarities, which are 10.2, 5.1, and 4.3 for water, methanol and ethanol, respectively [26]. In addition, silica surfaces are negatively charged and polar due to the presence of hydroxyl groups on the surface, which are crucial for interacting with the solvent upon loading [27]. Moreover, octreotide has 14 hydrogen bond donors and 16 hydrogen bond acceptors (S1), which caused water molecules to compete with octreotide to interact with the polar surface of silica and form hydrogen bonds with the negatively charged silanol groups. This reduced the loading efficiency. Methanol, on the other hand, is less polar than water, which caused octreotide to form more hydrogen bonds with the silanol groups and enhanced the loading efficiency [15]. In addition, the solubility of octreotide in methanol is higher than in water [28], which may have affected loading as it was based on reaching the equilibrium state followed by the solvent evaporation, leaving the drug in the pores [29]. Furthermore, the loading of octreotide with methanol does not impact the protein structure, as has been previously reported for the preparation of octreotide-loaded liposomes [30]. For samples loaded with ethanol, the low recovery was related to the solvent’s weak hydrogen bonding ability and the interactions between silanol groups on the surface, which reduced the loading efficiency [31].

BSA samples, on the other hand, showed lower recoveries compared to octreotide samples. The low values were attributed to several factors including protein aggregation, loading viscosity, solubility, protein molecular size, and diffusion which was the most prominent factor. However, the recovery was lower for methanol and ethanol than for water-based loading, where it was 32%, 10%, and 16% for water, ethanol, and methanol, respectively. This was related to the low solubility of BSA in both ethanol and methanol compared to water, where the solubility of BSA in alcohol-based solvents decreased with increasing solvent concentration [32]. In addition, ethanol caused BSA aggregation due to strengthened electrostatic interactions enhanced by the solvent [33].

Other factors contributing to differences in loading and recovery include viscosity, diffusivity, and protein molecular size.

### Effects of Viscosity and Protein Size on Diffusivity

Carrier loading is associated with the API molecules diffusing into the porous structure, which is governed by the Stokes-Einstein equation (Equation 1) where the particles are spherically shaped [34].

$$D_{0}=\frac{k_{B\;}\theta }{6\eta \pi R_{m}}$$

Where K_B_ is the Boltzmann constant, θ is the temperature, η is the viscosity, and R_m_ is the solute radius. The previous equation could be used to determine protein diffusivity in solutions [35], where it is disproportionate to the radius and viscosity. According to Erickson [36], the protein radius (R_m_) could be obtained, assuming that it will take a spherical shape, by calculating the volume occupied by protein mass (V) as seen in Equation 2:

$$V=1.212*10^{-3}*M$$

Where M is the molecular weight in Da. After calculating the protein mass, R_m_ can be calculated as shown in Equation 3 below:

$$R_{m}=\sqrt[{3}]{\left(\frac{3V}{4\pi }\right)}$$

The viscosities of the solvents were 0.55, 0.89, and 1.096 mPa.s for methanol, water and ethanol, respectively [37, 38]. As mentioned above, the viscosity of the loading solvent affected the mass transfer between the solvent and the loaded system. Moreover, the viscosity of the loading phase was calculated and presented in Table 1:

**TABLE 1:** Dynamic viscosity values of the loading solvents containing BSA and octreotide at different loading concentrations.

Solvent	Octreotide	BSA
Methanol	5.92*10^–10^	1.47*10^–10^
Water	3.66*10^–10^	9.13*10^–11^
Ethanol	2.97*10^–10^	6.73*10^–11^

By applying Equations 1, 2, the radius for BSA and octreotide were 2.69 and 0.67 nm, respectively, and the diffusivity values are presented in Table 2:

**TABLE 2:** diffusivity values (m2/s) of BSA and octreotide in loading solvents.

Dynamic viscosity (mPa.s)						
Concentration	Octreotide			BSA		
	Water	Methanol	Ethanol	Water	Methanol	Ethanol
5% w/w	1.12 ± 0.00	0.69 ± 0.00	1.27 ± 0.01	1.12 ± 0.04	0.7 ± 0.01	1.30 ± 0.00
10% w/w	1.08 ± 0.01	0.65 ± 0.01	1.26 ± 0.02	1.32 ± 0.01	0.71 ± 0.00	1.34 ± 0.03
20% w/w	1.11 ± 0.00	0.68 ± 0.02	1.26 ± 0.01	1.37 ± 0.04	0.71 ± 0.01	1.30 ± 0.01

As shown in Tables 1, 2, the diffusivity of octreotide in methanol was the highest (5.926*10^–10^ m^2^/s), due to the viscosity of both the methanol and methanol-octreotide phases and the high solubility of octreotide (40 mg/mL). For BSA, the diffusivity was lowest when ethanol was the loading solvent (6.731*10^–11^ m^2^/s). This was related to the low solubility of BSA in ethanol and the large radius (2.69 nm). These findings support the HPLC recovery results, where octreotide formulations with methanol had the highest recovery percentage.

The protein dispersions in the loading solvents were analysed using laser diffraction and dynamic light scattering for dissolved proteins (octreotide in all solvents and BSA in water) to further investigate protein aggregation.

Octreotide was completely dissolved in the three solvents, which is consistent with its high solubility [28]. As for BSA, it had dissolved in water, which was expected considering its high solubility in water [39]. However, BSA did not dissolve in either methanol or ethanol regardless of its concentration, and the particle sizes were 223.63 and 231.06 μm in ethanol and methanol, respectively. This confirms that BSA aggregates at high concentrations and HPLC results, where the recovery of 5% w/w loading was 0% and for 20% it was 10% and 16% for ethanol and methanol, respectively [40].

### Molecular Interactions of the SYLOID-Proteins

FTIR was used to determine the molecular interactions and the presence of both proteins and the spectrum for both SYLOID-octreotide and SYLOID-BSA formulations (Supplementary Figures S2, S3).

For the spectrum of SYLOID (S2), a broad band at 1075 cm^−1^ corresponded to the asymmetric stretching of the siloxane group (Si-O-Si). In addition, the peaks at 800 cm^−1^ and 970 cm^−1^ corresponded to the symmetric stretching vibration of Si-O and Si-OH, respectively [41]. For octreotide, both the C=O and -NH groups corresponded to peaks at 1650 and 1540 cm^−1^, respectively, indicating the presence of octreotide [42]. Moreover, the decrease in intensity of the broadband at 1075 cm^−1^ for methanol-based samples indicated an interaction between the siloxane groups and octreotide, which could have masked the signal. This further supported the fact that octreotide had diffused and formed new bonds with siloxane groups from methanol more efficiently than from ethanol and water. In formulations containing BSA, (S3), the carbonyl group corresponded to a strong peak at 1650 cm^−1^, while the bands at around 1375 and 1450 cm^−1^ corresponded to CH and CH2 [43]. Moreover, the stretching of the amide bond at 1670 cm^−1^ indicated that BSA had been adsorbed on the surface of the silica carrier.

Fluorescence imaging was implemented to further evaluate the protein diffusivity and diffusion into the silica carrier.

### Fluorescence Properties of Proteins and Diffusion in Silica Carriers

Confocal microscopy has been reported to study the diffusion of proteins into silica carriers based on fluorescence intensity by Lu et al [35]. The presence of tryptophan in the structure of both BSA and octreotide has assisted in the identification of fluorescence due to its presence in both proteins [44, 45].

**FIGURE 3:**
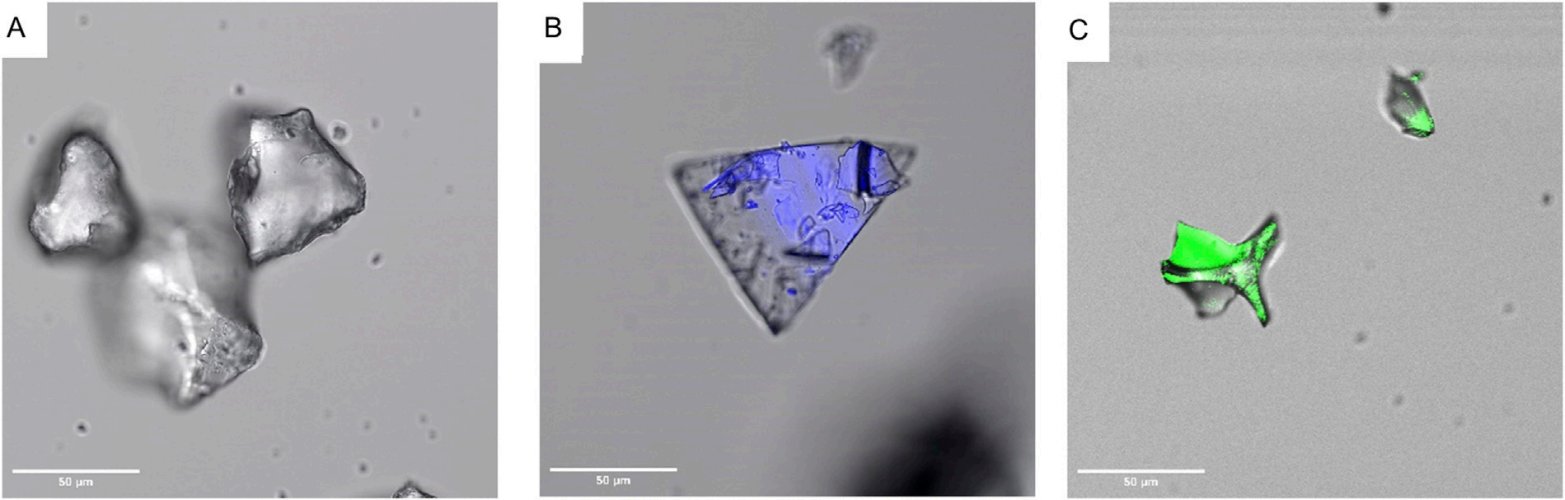
Fluorescence behaviour of peptides and silica by confocal microscopy where **(A)**: SYLOID XDP, **(B)**: octreotide, **(C)**: BSA.

Figures 3A–C shows plain SYLOID, octreotide and BSA, respectively, where SYLOID showed no fluorescence while octreotide (blue) and BSA (green) were fluorescent.

**FIGURE 4:**
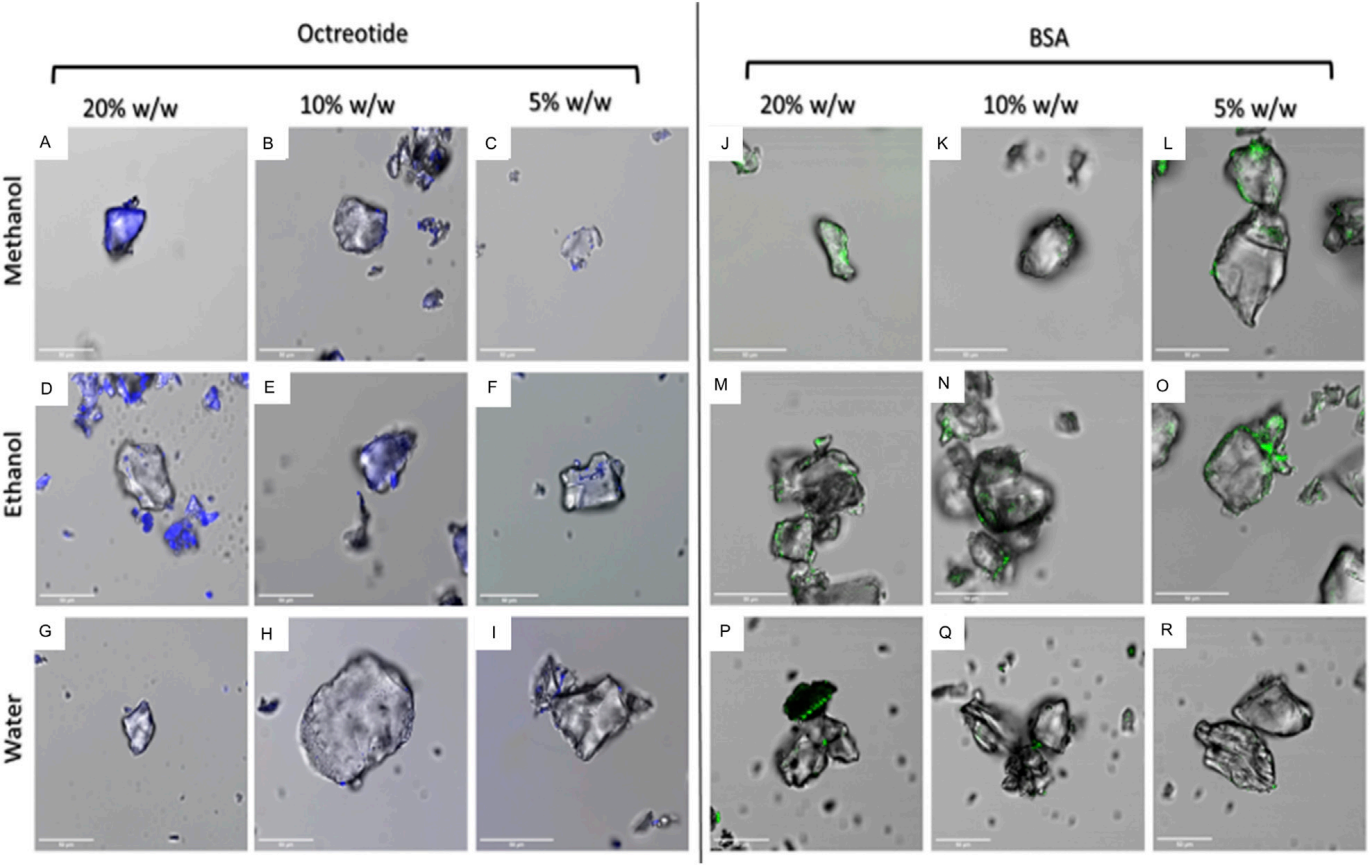
Fluorescence properties of peptide-silica formulations in different solvents and amounts (w/w). octreotide loaded in methanol **(A)**: 20%, **(B)**: 10%, **(C)**: 5%, loaded in ethanol **(D)**: 20%, **(E)**: 10%, **(F)**: 5%, and water loading **(G)**: 20%, **(H)**: 10%, **(I)**: 5%. BSA loaded in methanol **(J)**: 20%, **(K)**: 10%, **(L)**: 5%, ethanol loading **(M)**: 20%, **(N)**: 10%, **(O)**: 5%, and loaded in water **(P)**: 20%, **(Q)**: 10%, **(R)**: 5%.

Figure 4 below shows the surface fluorescence of all formulations. It can be seen that octreotide samples Figures 4A–I showed more surface fluorescence compared to BSA Figures 4J–R. Moreover, octreotide samples created with methanol had a higher fluorescence than those created with ethanol and water, which could be seen in Figures 4A–C compared to Figures 4D–F. In addition, the surface fluorescence appeared to increase as the loading concentration increased. For instance, octreotide loaded in methanol at a loading concentration of 20% w/w Figure 4A showed higher surface fluorescence in comparison to lower loading concentrations: Figures 4B, C Moreover, octreotide loaded via water had the lowest surface fluorescence properties Figures 4G–I, with 5% loading having the lowest fluorescence among the octreotide formulations Figure 4I.

**FIGURE 5:**
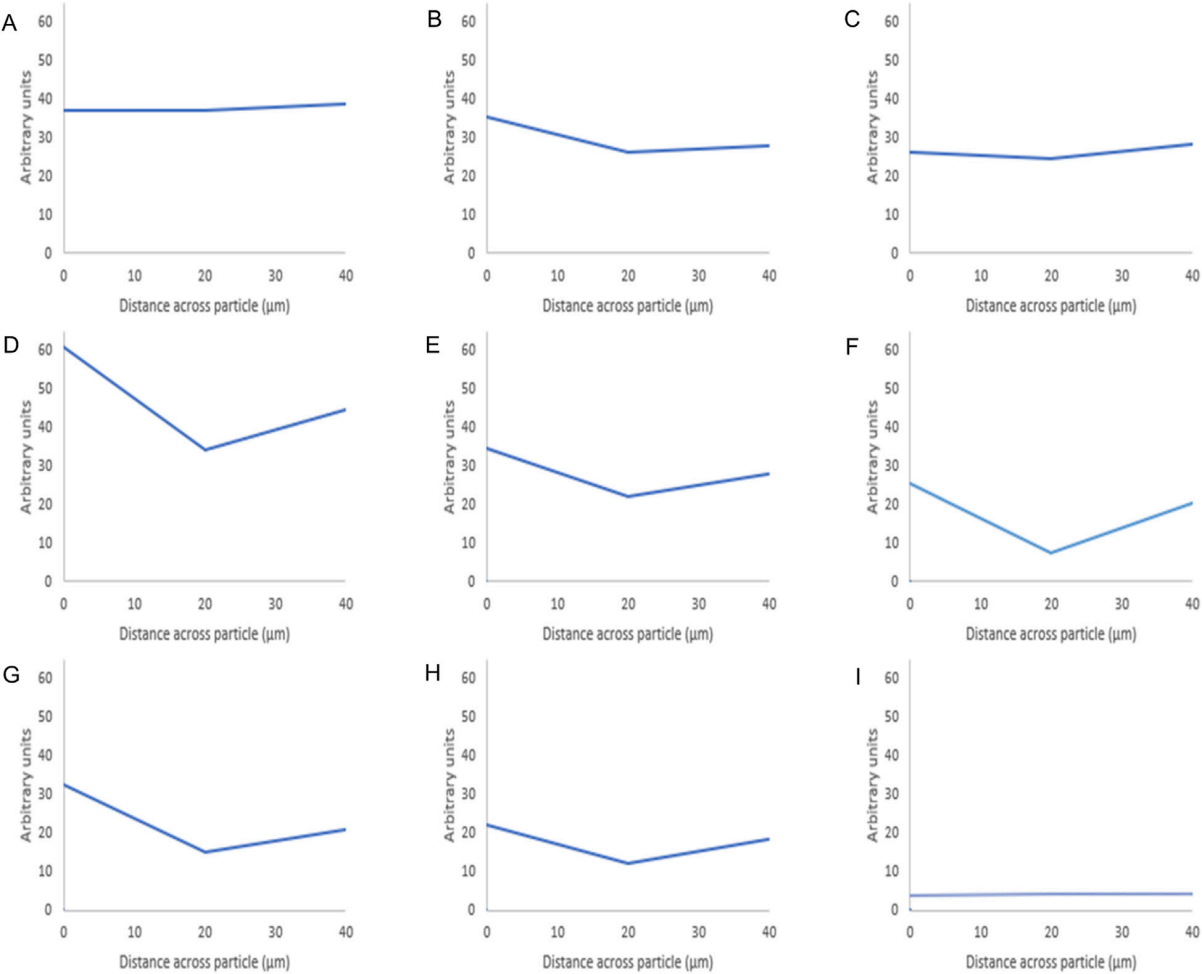
Fluorescence intensity values of octreotide-silica formulations. The intensity value for each particle is the average intensity of 10 particles where the (Y) axis represents the arbitrary units and the (X) axis resembles the distance taken across the middle plane of the particle after 3D imaging. The first row shows the methanol-based loading: 20% **(A)**, 10% **(B)**, 5% **(C)**, while the second row shows the ethanol-based loading: 20% **(D)**, 10% **(E)**, 5% **(F)**, and the third row is the water-based loading 20% **(G)**, 10% **(H)**, 5% **(I)**.

**FIGURE 6:**
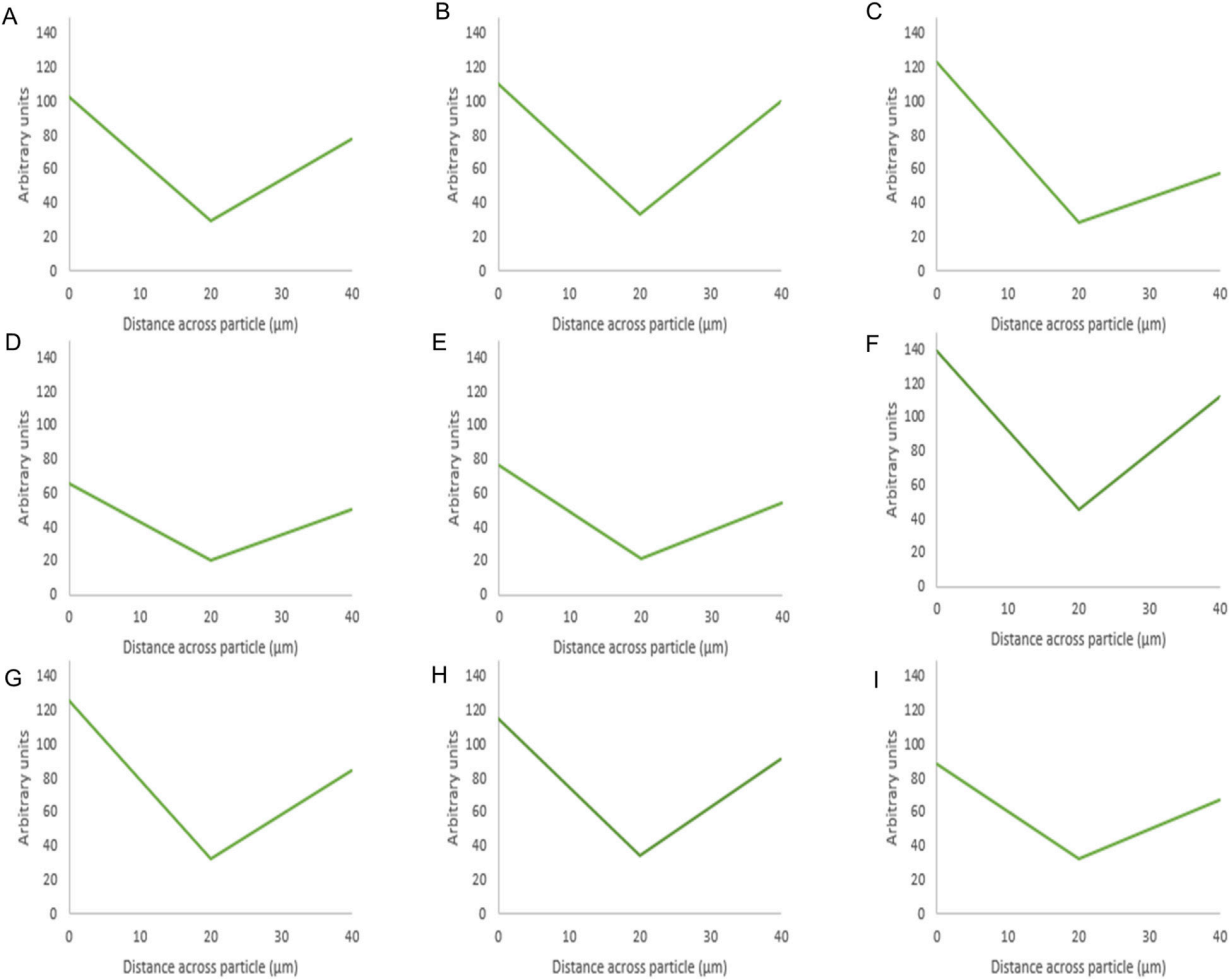
Fluorescence intensity values of BSA-silica formulations. The intensity value for each particle is the average intensity of 10 particles where the (Y) axis represents the arbitrary units and the (X) axis resembles the distance taken across the middle plane of the particle after 3D imaging. The first row shows the methanol-based loading: 20% **(A)**, 10% **(B)**, 5% **(C)**, while the second row shows the ethanol-based loading: 20% **(D)**, 10% **(E)**, 5% **(F)**, and the third row is the water-based loading 20% **(G)**, 10% **(H)**, 5% **(I)**.

For BSA, BSA samples seemed to have lower surface fluorescence Figures 4J–R compared to octreotide samples. Furthermore, it appeared that the protein was concentrated on the sides of the silica carrier and the fluorescence intensity decreased as the loading concentration decreased Figures 4J–L. These results gave a preliminary idea of how the protein interacted with the carrier. The fluorescence intensity values of the middle plane from the generated 3D images were calculated and presented in Figures 5, 6 below.

As seen in Figure 5 below shows that when using methanol as the loading solvent, octreotide diffused equally into the silica particle at 20% w/w Figure 5A. Upon reducing the loading concentration, the fluorescence intensity decreased, but the protein diffused thoroughly into the carrier Figures 5B, C. However, when ethanol and water were used, octreotide seemed to have inhabited the sides of the carrier in a low diffusion manner with respect to the loading concentrations. In addition, water showed very little diffusion into the centre of the carrier been crystallised on the loading concentration was 5% w/w. The difference in diffusivity was related to the solvent viscosity and the peptide solubility. For instance, methanol had the lowest viscosity and presented the highest diffusivity, as calculated above (5.926*10^–10^), compared to water (3.662*10^–10^) and ethanol (2.973*10^–10^). Additionally, it has been reported that octreotide has a solubility of 40, 10.04, and 28.85 mg/mL in methanol, ethanol and water, respectively [28]. The low solubility of octreotide in ethanol and the highly polar properties of water affected the loading efficiency. These results further support the recovery results, with methanol showing the higher percentage at 20% loading w/w, while water showed the lowest recovery (3%) at a 5% loading concentration.

For BSA, the protein appeared to be present at the edges as the fluorescence intensity values were high around the sides of the silica particle. This was evident for the three solvents as the protein appeared to mostly occupy the edges upon loading using ethanol at 20% w/w Figure 6G. The presence of protein at the edges was related to its low solubility in ethanol and methanol and the effects of the previous solvents on its aggregation as mentioned above. In addition, the larger radius of BSA (2.69) compared to octreotide (0.67 nm) contributed to these observations as BSA would struggle to occupy SYLOID’s non-ordered pore structures.

These findings support the results presented earlier regarding the recovery percentage, with octreotide formulations and in particular methanol-based loading, having the highest recovery. Nitrogen porosimetry was used to further corroborate how proteins interact with the carrier and diffuse, which is explained in the following *Effects Of Loading Parameters on Surface Area and Pore Volume*.

### Effects of Loading Parameters on Surface Area and Pore Volume

Surface area and pore volume were calculated for the peptide-silica formulations to assess the effects of loading solvent and concentration on them. Table 3 below shows the surface area and pore volume of the formulations.

**TABLE 3:** Surface area and pore volume of peptide-silica formulations.

Loading solvent	Initial loading concentration (w/w) (%)	Octreotide		BSA	
		Surface area (m^2^/G)	Pore volume (cc/g)	Surface area (m^2^/G)	Pore volume (cc/g)
Water	5	283.89	1.77	229.82	1.38
	10	258.18	1.48	210.62	1.31
	20	208.22	1.41	170.82	1.17
Methanol	5	224.87	1.42	271.09	1.71
	10	231.61	1.38	246.40	1.36
	20	203.53	1.20	193.02	1.48
Ethanol	5	225.80	1.38	216.98	1.44
	10	211.38	1.35	245.20	1.43
	20	217.55	1.24	215.16	1.24

SYLOID XDP 3050 is a non-ordered mesoporous silica carrier that has a multi-directional and non-uniform pore structure. It has a pore volume of 1.75 cc/g and a surface area of 285 m^2^/g, as reported in the literature [46]. Moreover, the pore network is non-homogenous and contains interconnected pores [47, 48]. The table below shows that the pore volume decreased as the loading concentration increased for all solvents. In addition, for octreotide-based formulations, the lowest pore volume corresponded to a methanol loading at 20% w/w, meaning that the drug had occupied the porous structure more efficiently than other solvents. Furthermore, the low pore volume of methanol loading samples supported the fluorescence intensity results, where the protein had diffused evenly into the carrier. In addition, the highest calculated pore volume corresponded to the water-based formulation at a loading concentration of 5%. This was related to the polar properties of water and the competition with octreotide to interact with the silica groups on the surface. Additionally, the surface area decreased with the loading concentration increase, which may be related to the protein adsorbed on the silica surface.

For BSA samples, BSA loaded in methanol at 5% w/w showed the highest pore volume of 1.71 cc/g, which was related to the low amount of the drug inside the silica carrier. However, the lowest pore volume was associated with water-based loading at 20% w/w, which contributed to the drug inside the porous structure. In addition, ethanol-based loading formulations showed a similar observation trend the pore volume decreased as the loading concentration increased. Even though BSA has very low solubility in ethanol, some crystals may have inhabited the porous structure of SYLOID and decreased the pore volume as a result. The surface area results presented a similar pattern to the octreotide formulations, where it decreased as the drug loading concentration increased, which may be related to the drug adsorbing on the surface, and decreasing the surface area.

### Morphological Properties of the Carrier and the Peptide Silica Complex

**FIGURE 7:**
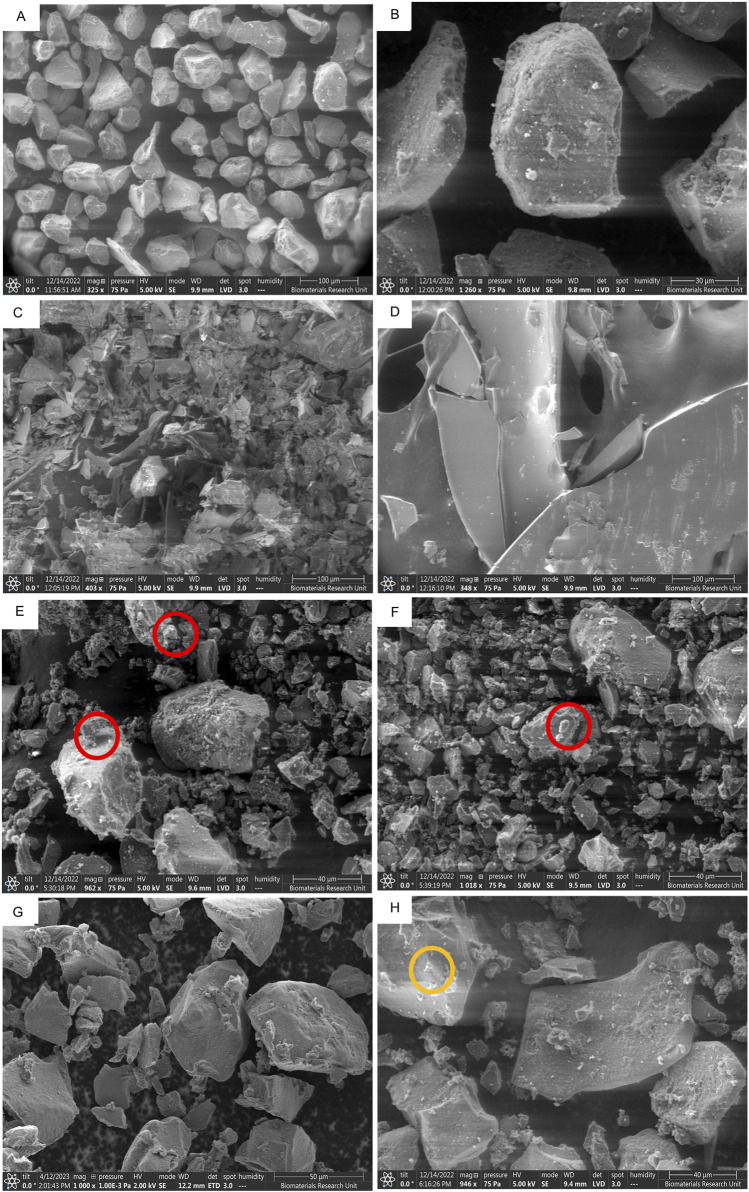
SEM images of different peptide-silica complexes showing surface morphology and protein crystal adherence. SYLOID XDP 3050 low magnification **(A)**, and high magnification **(B)**, octreotide **(C)**, BSA **(D)**, octreotide 20% in methanol **(E)**, octreotide 20% in ethanol **(F)**, octreotide 20% in water **(G)**, and BSA 20% in water **(H)**. The red and orange circles highlight protein crystals adhering to the surface for octreotide and BSA * BSA samples in methanol and ethanol were not imaged due to the low solubility of the protein in both solvents.

The formation of crystals on the silica carrier and the difference in morphological features can be seen in Figures 7A–H, where silica Figures 7A, B, octreotide Figure 7C, and BSA Figure 7D were imaged as a reference for comparison.

SYLOID 3050 is a non-ordered mesoporous silica where the pore size varies between particles Figure 7A. The surface of the particle is usually smooth Figure 7B. Octreotide presented a crystalline morphology under the microscope Figure 7C, compared to the large crystals of BSA Figure 7D, which was supported by the literature [49].

Figures 7E–G represented depict 20% w/w loading in methanol, ethanol and water, respectively, while Figure 7H depicts BSA 20% w/w loading in water. As can be seen from the figure above, small crystal structures were adhered to the surface of the silica surface in Figure 7E, F (red circles), while in Figure 7G they did not appear in comparison to the plain carrier Figure 7B. This was related to octreotide crystallising on the surface from both methanol and ethanol due to the low polar properties of both solvents compared to water Figure 7G. These crystal-like structures contributed to the high recovery from both solvents, as discussed above, where water showed the lowest recovery, and further supported the fluorescence intensity results, where water-based loading showed the lowest fluorescence intensity. On the other hand, the BSA formulation Figure 7H contained small crystals that adhered to the surface (orange circle), which may be related to the small amount of BSA being adsorbed.

## Conclusion

We have created peptide-silica complexes using SYLOID microparticles by solvent evaporation, while investigating factors affecting BSA and octreotide loading, such as solvent polarity, viscosity, protein size, and diffusivity. Protein loading into SYLOID was based on diffusion, governed by the Stokes-Einstein equation, and affected by the molecular size of the protein and solvent viscosity. Octreotide had the highest recovery (71%) and greater diffusion using methanol due to its polar properties and protein solubility. The diffusivity of octreotide was approximately 6.5 times higher than that of BSA, while ethanol and methanol decreased the diffusion of BSA by increasing the dispersed particle size. 3D confocal fluorescence imaging revealed that octreotide diffused into the carrier, while BSA was concentrated at the edges of the particle. Furthermore, SEM imaging identified the presence of protein crystallising on the surface, with BSA found to be crystallising on the particle surface. Pore size analysis showed that methanol loading for octreotide had the smallest pore volume, indicating diffusion of the protein into the porous structure. This study highlights the importance of considering protein size, solvent properties, and diffusion characteristics when utilising silica-based carriers for protein delivery. Understanding these factors can contribute to the development of more effective oral protein-based therapeutics where the release and permeability of proteins from the SYLOID- based complex will be investigated in future work.

## Summary Table

### What Is Known About This Subject

• Mesoporous silica is a great candidate for loading biologics due to its large pore volume and surface area.
• Numerous loading methods exist and they are affected by the properties of the molecule and the solvent.

### What This Paper Adds

• The loading of biologics is governed by diffusion and is based on the Einstein-Stokes equation.
• 3D fluorescence intensity imaging identifies diffusion patterns based on protein positioning.
• Solvent polarity affects diffusivity as methanol presents even diffusion for octreotide.

## Concluding Statement

This work represents an advance in biomedical science because it correlates between solvent and protein properties for the successful loading of silica microparticles intended for oral delivery.

## Data Availability

The original contributions presented in the study are included in the article/Supplementary Material, further inquiries can be directed to the corresponding authors.

## Supplementary Material

SUPPLEMENTARY FIGURE S1 Chemical structures of octreotide acetate and BSA.

SUPPLEMENTARY FIGURE S2 Spectrum of octreotide-silica formulations for the three solvents at a loading concentration of 20% w/w.

SUPPLEMENTARY FIGURE S3 Spectrum of BSA-silica formulations for the three solvents at a loading concentration of 20% w/w.

## Abbreviations

API, active pharmaceutical ingredient; BSA, bovine serum albumin; GI tract, gastrointestinal tract; GRAS, generally recognised as safe.
