# Supplementary figures and images for: Mesoporous Silica Microparticle-Protein Complexes: Effects of Protein Size and Solvent Properties on Diffusion and Loading Efficiency
Source: Br J Biomed Sci. 2024 Oct 9;81:13595. doi: 10.3389/bjbs.2024.13595 (PMC11496099; doi:10.3389/bjbs.2024.13595)

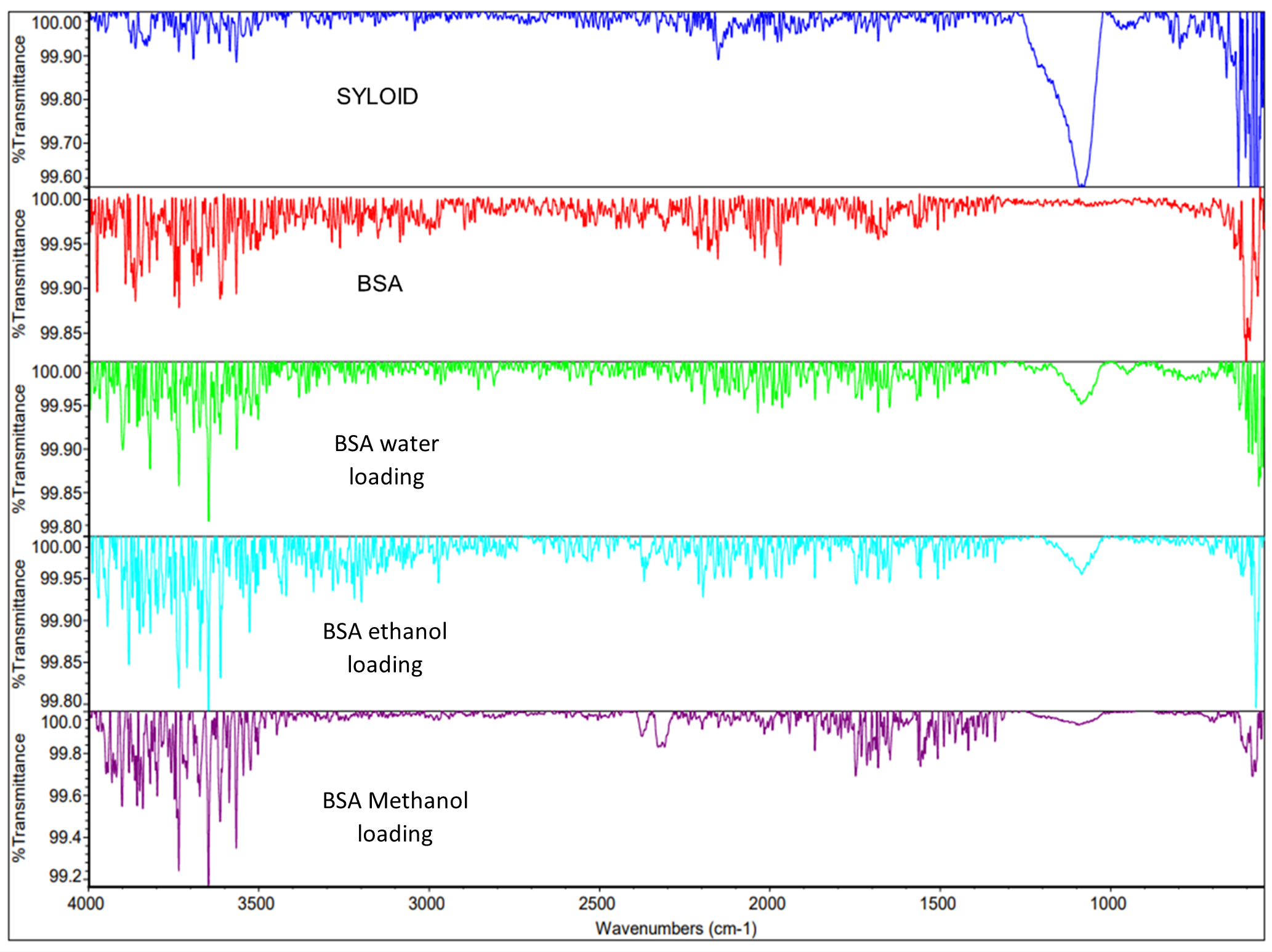

Supplement: Supplementary file 1 [file Image3.TIF]

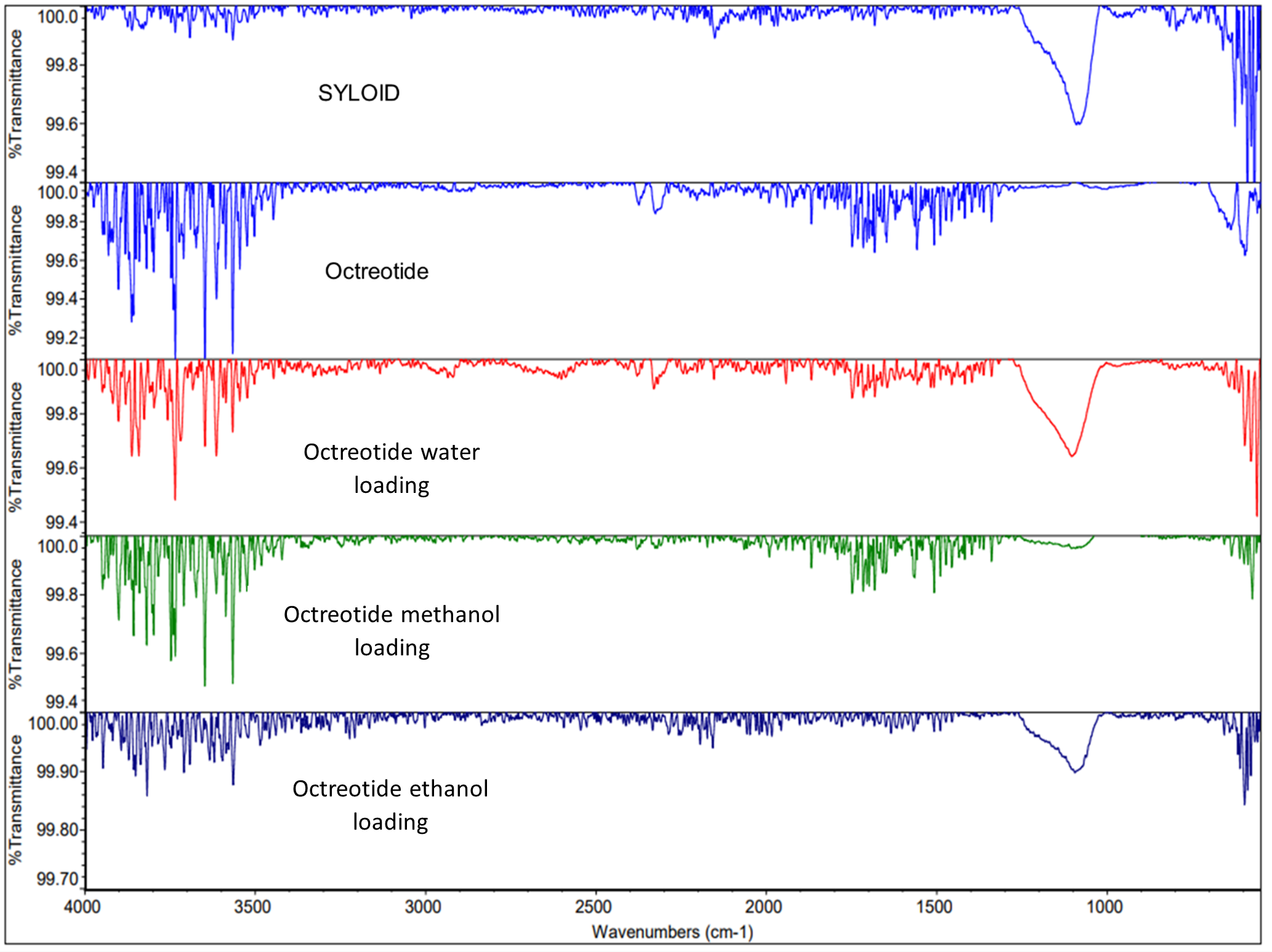

Supplement: Supplementary file 2 [file Image2.TIF]

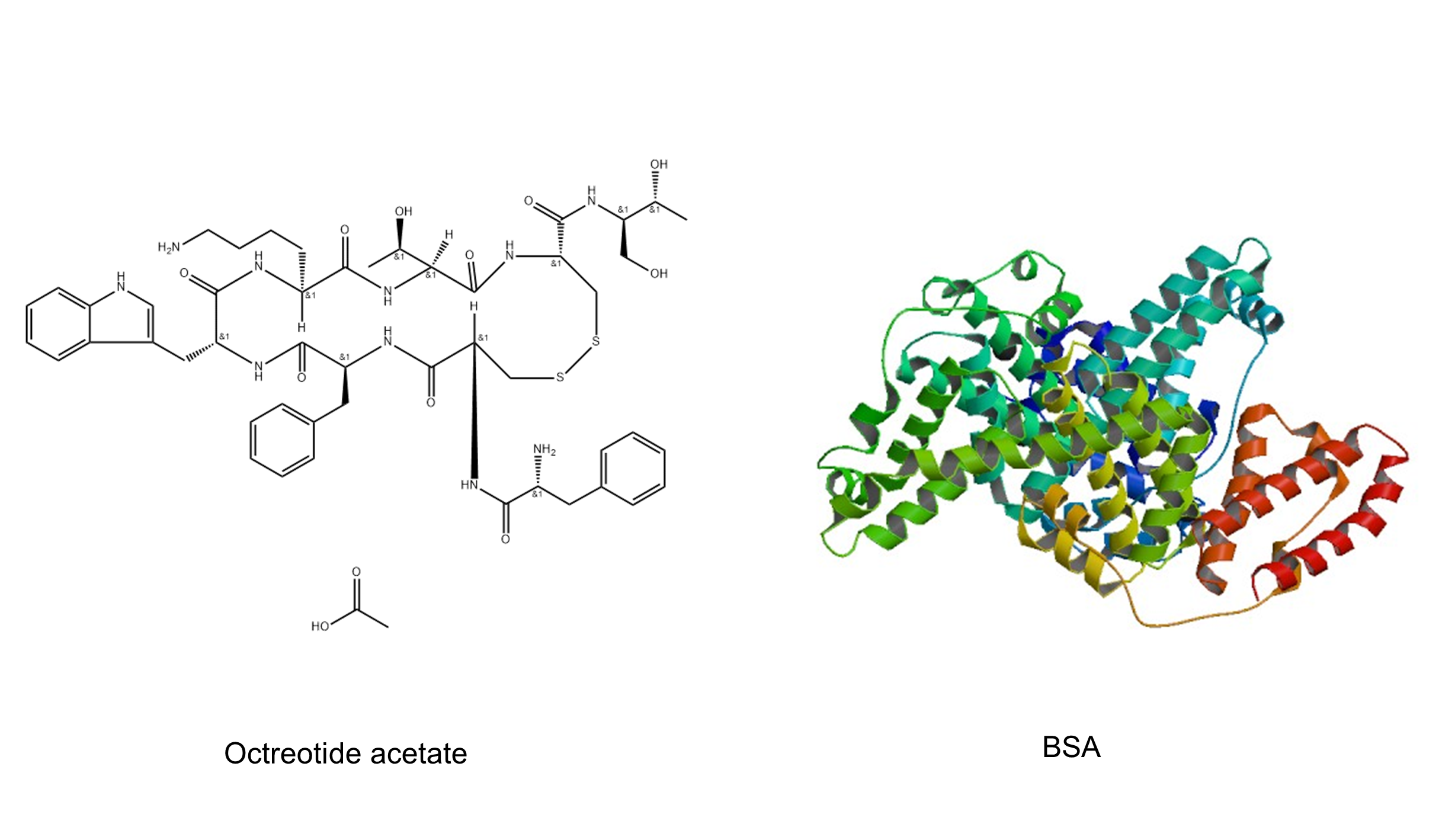

Supplement: Supplementary file 3 [file Image1.TIF]
